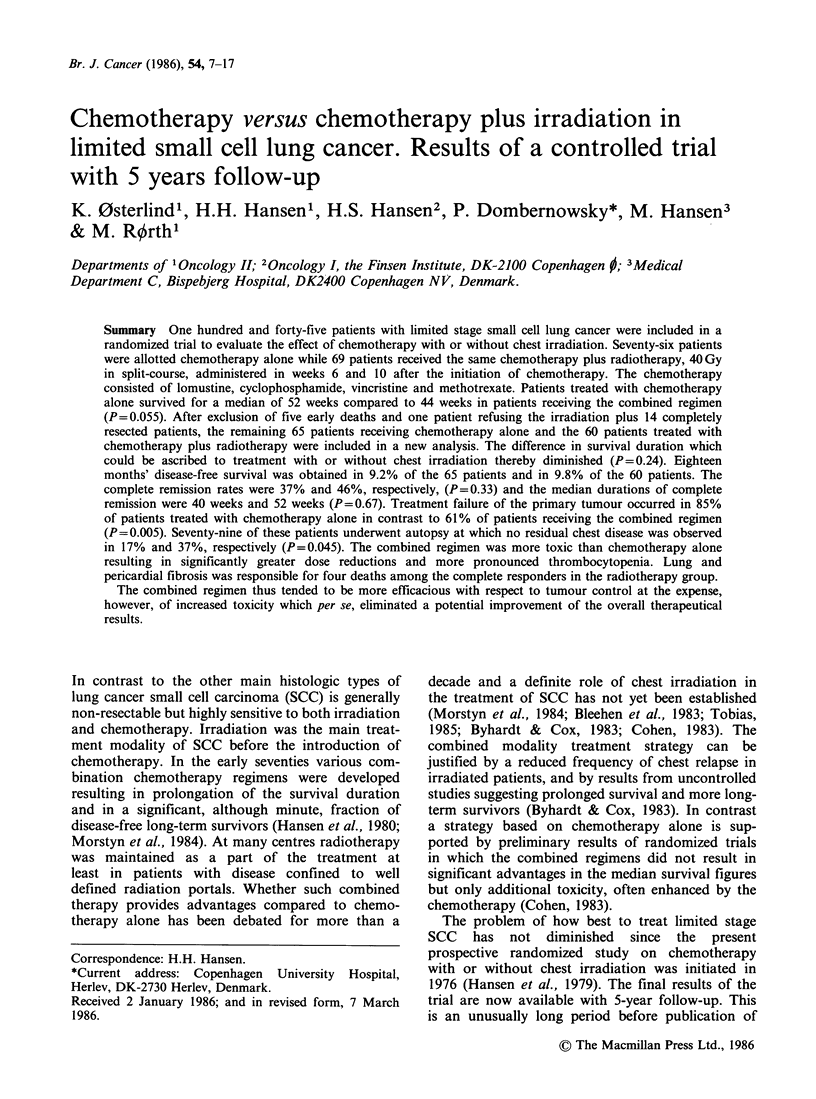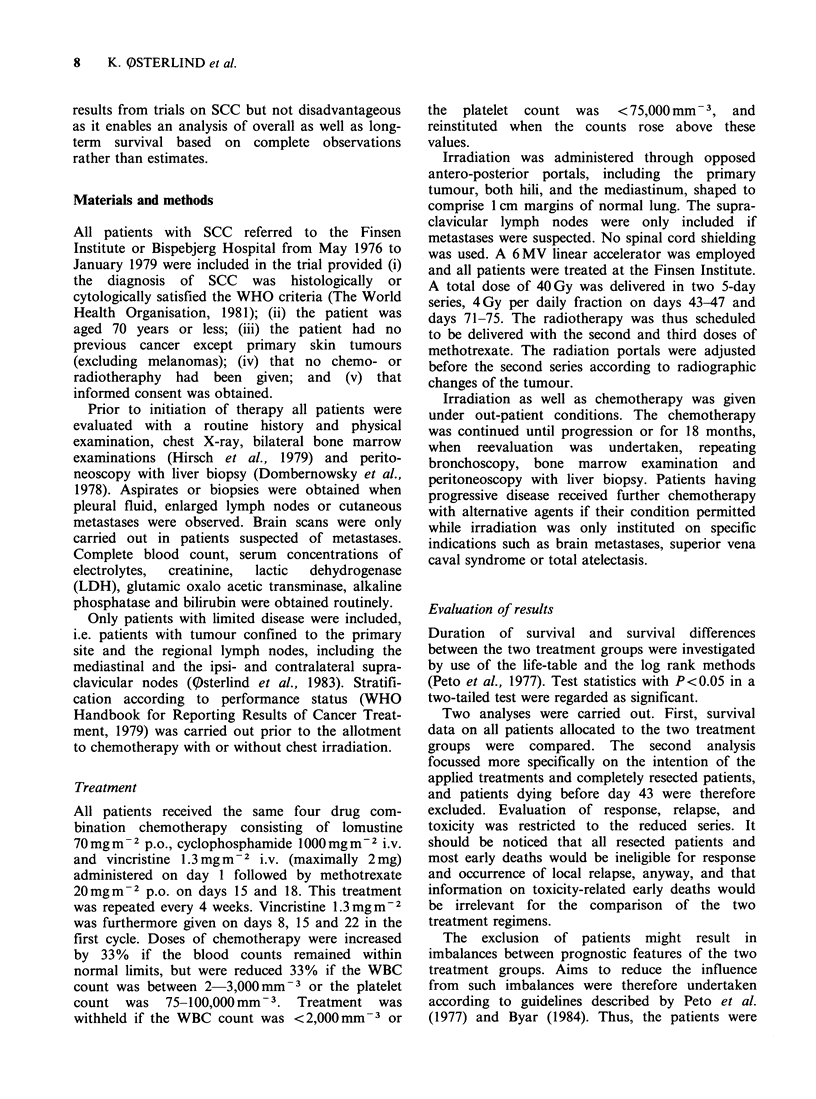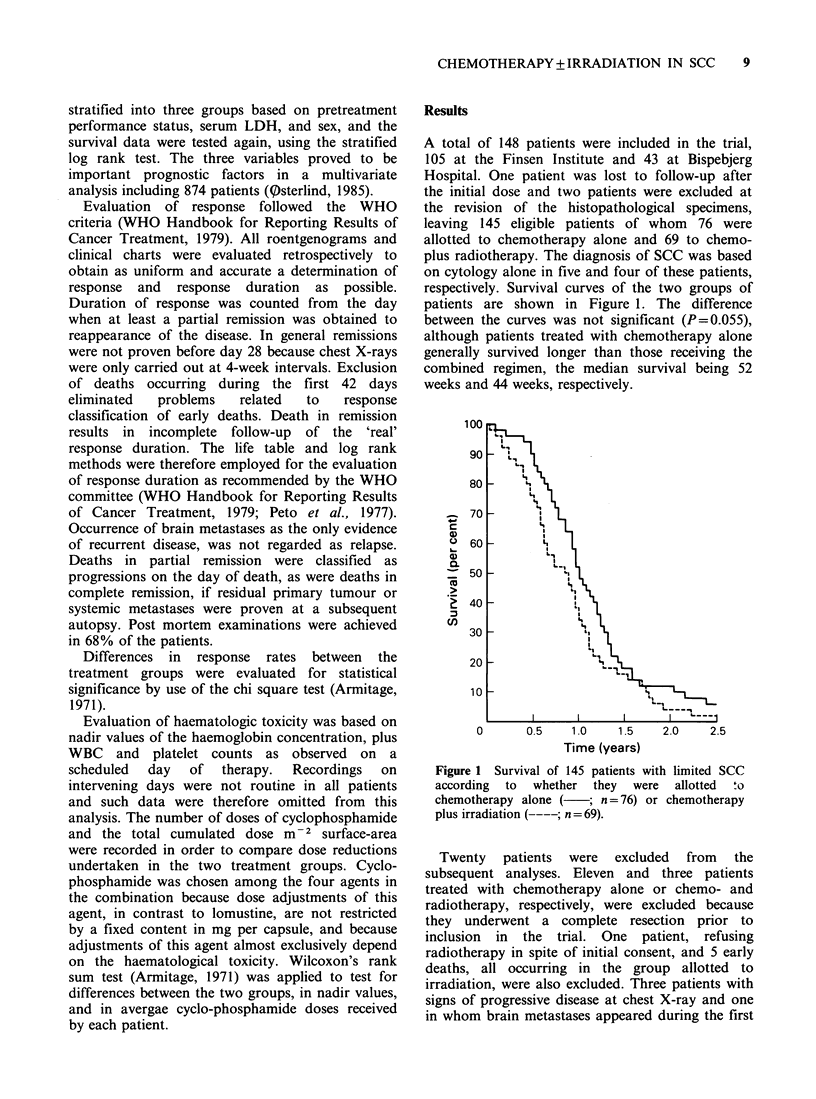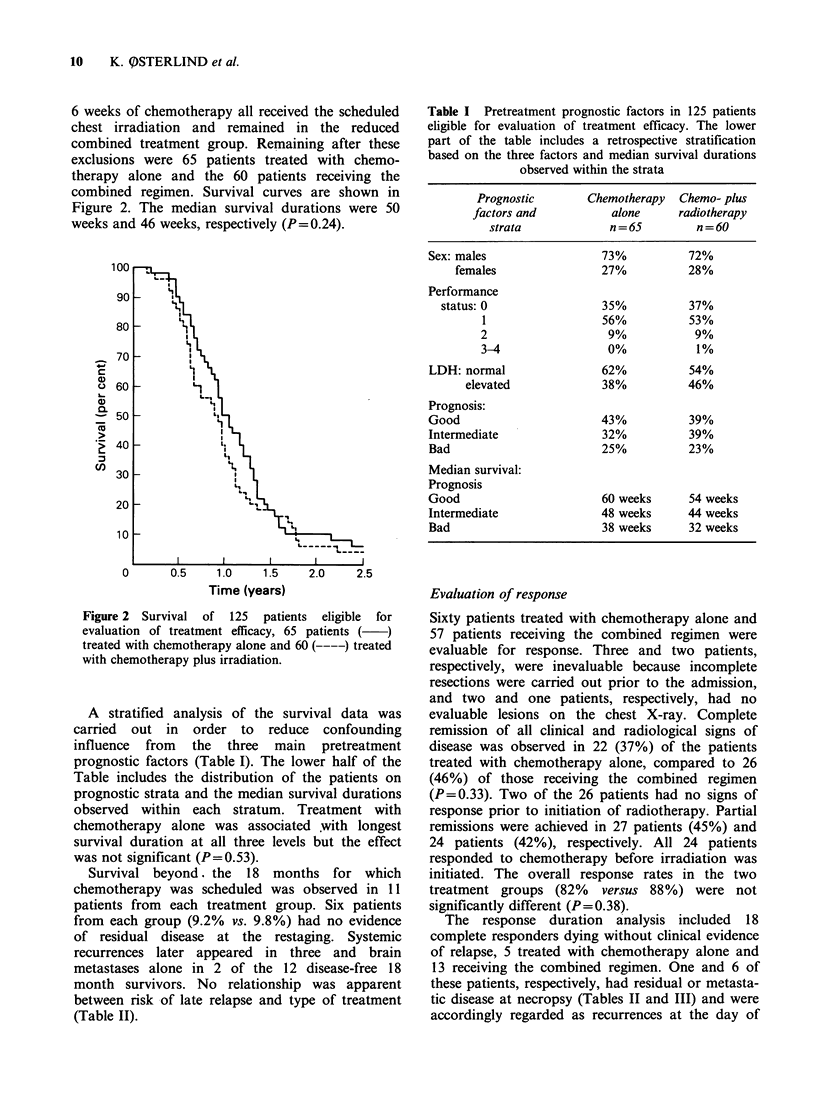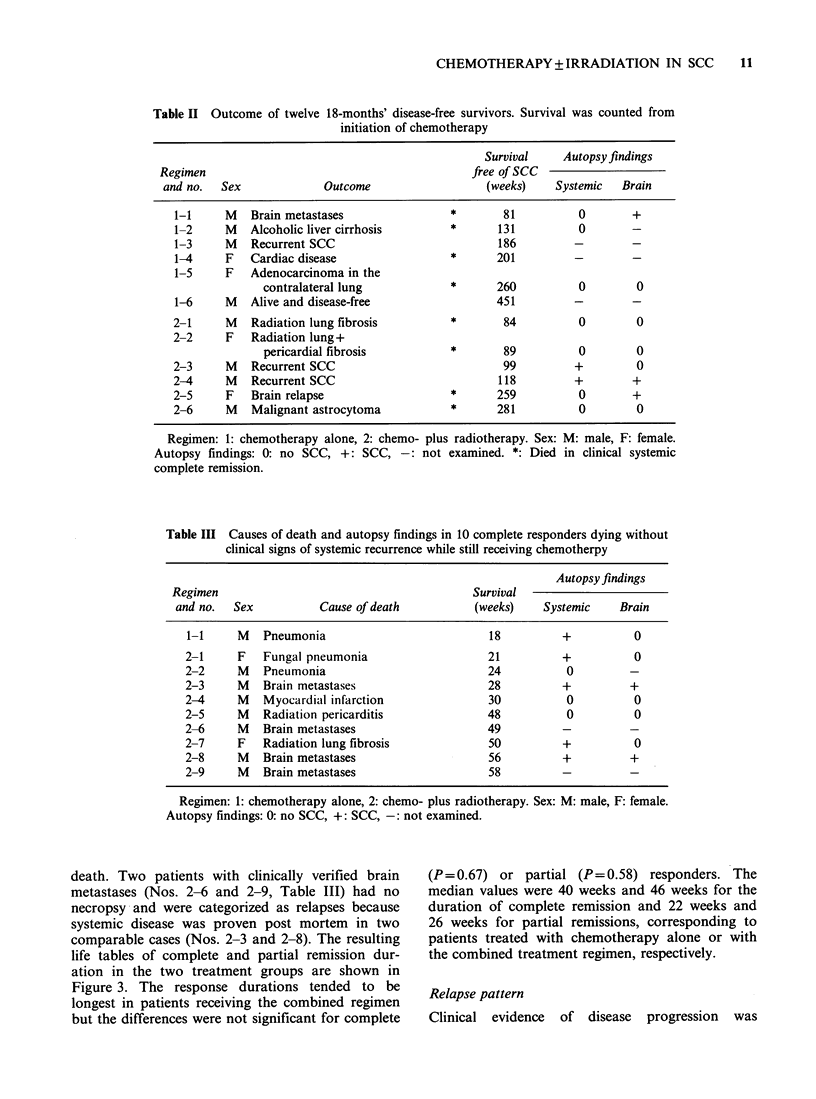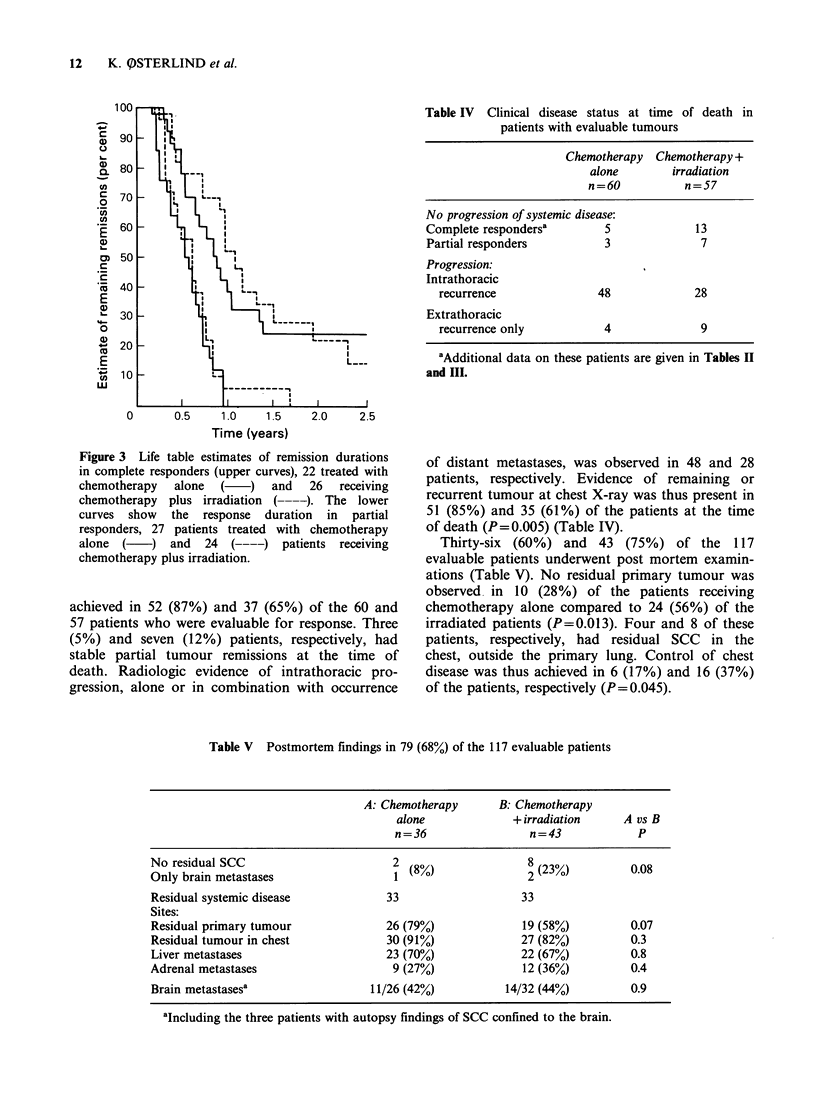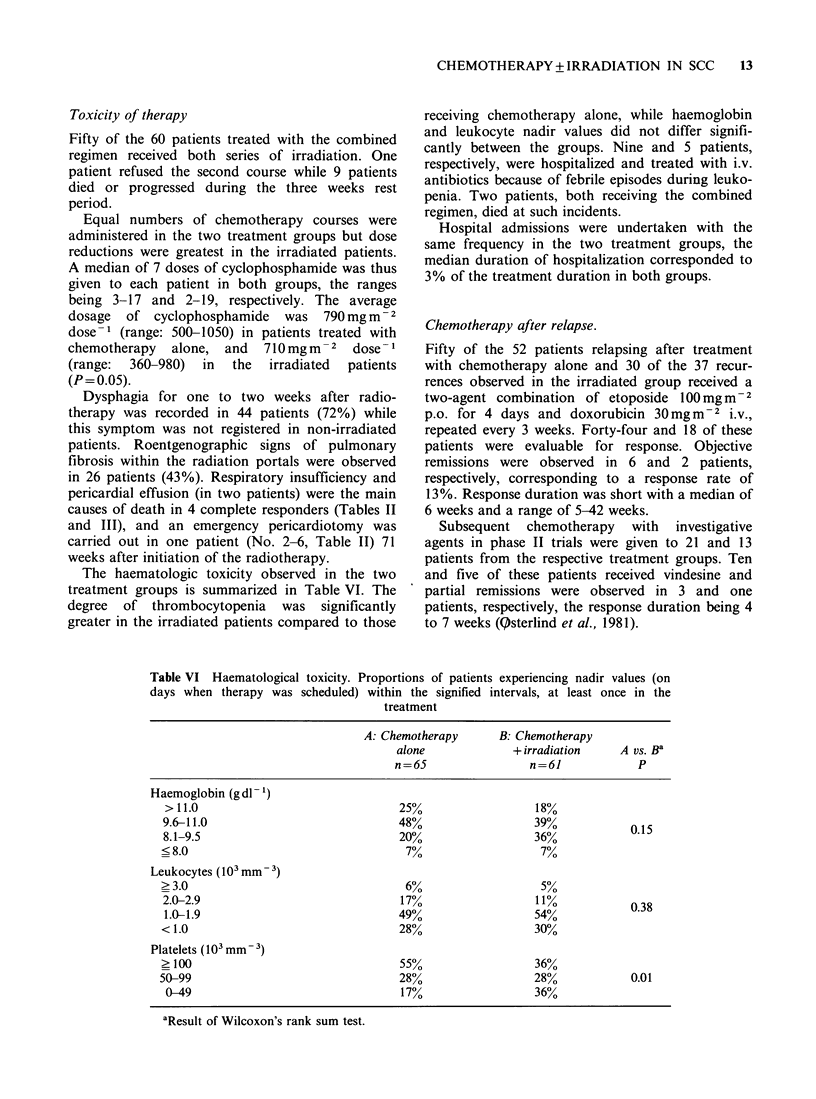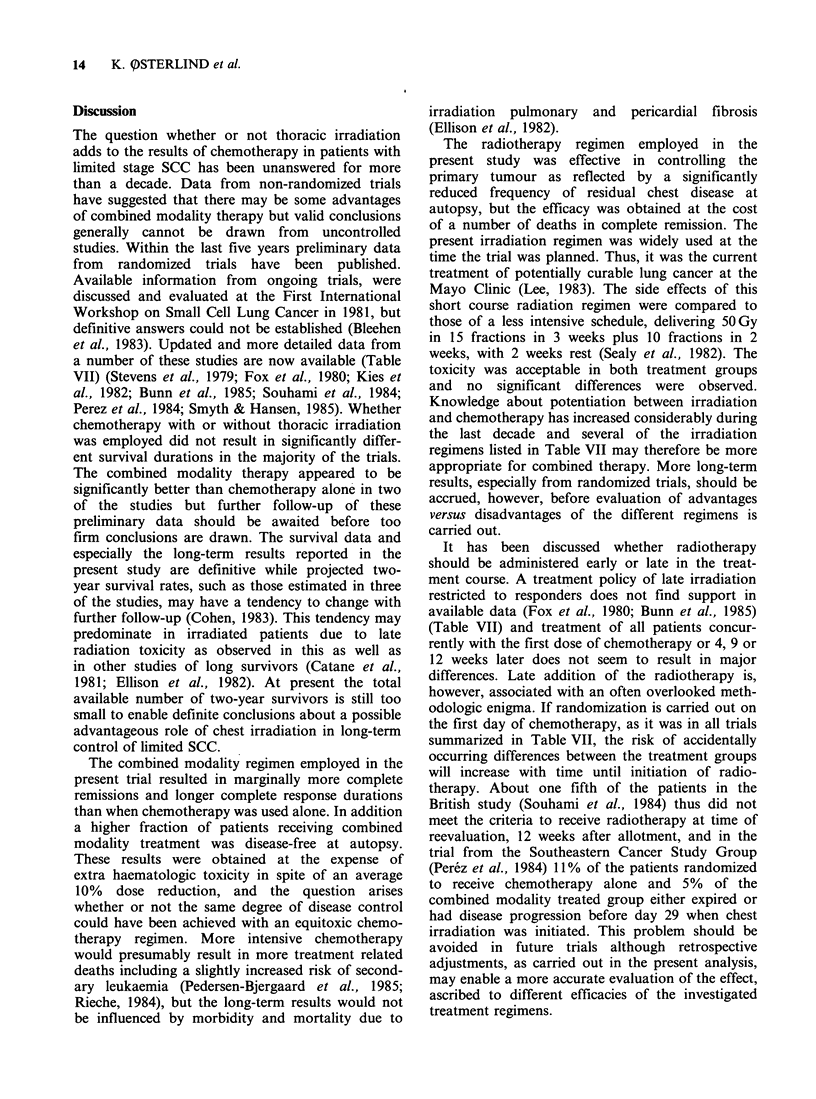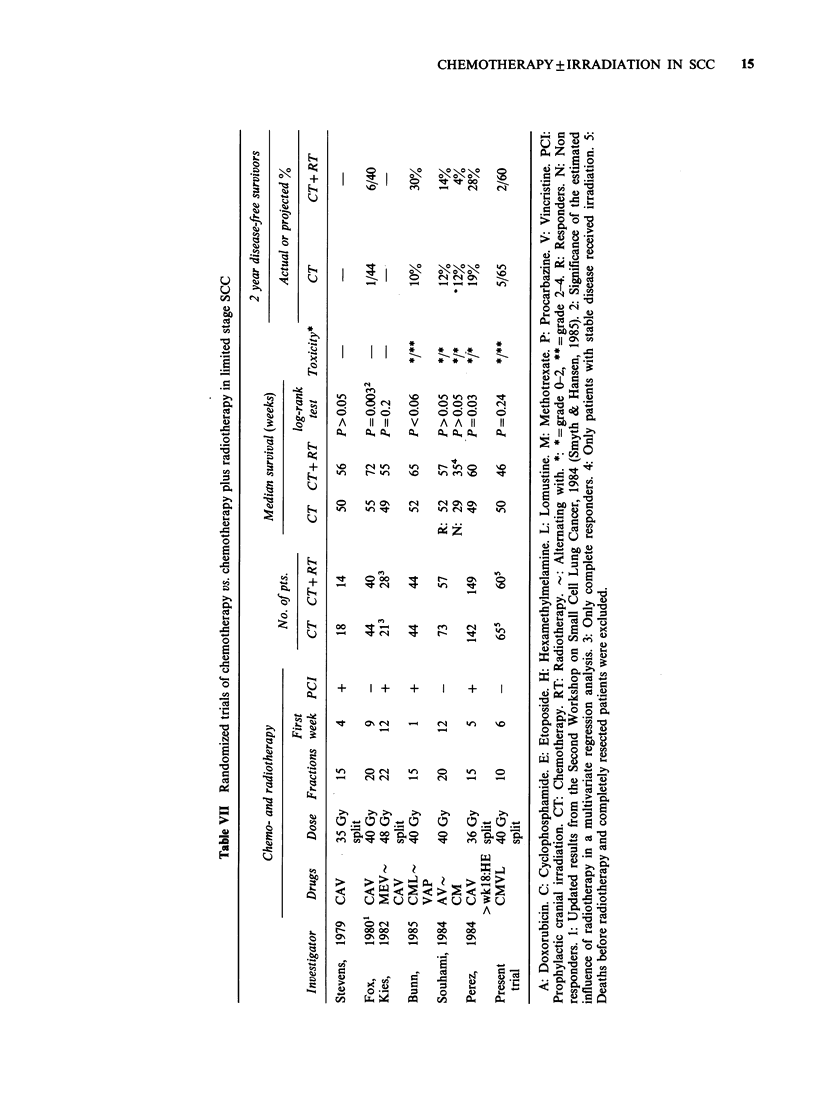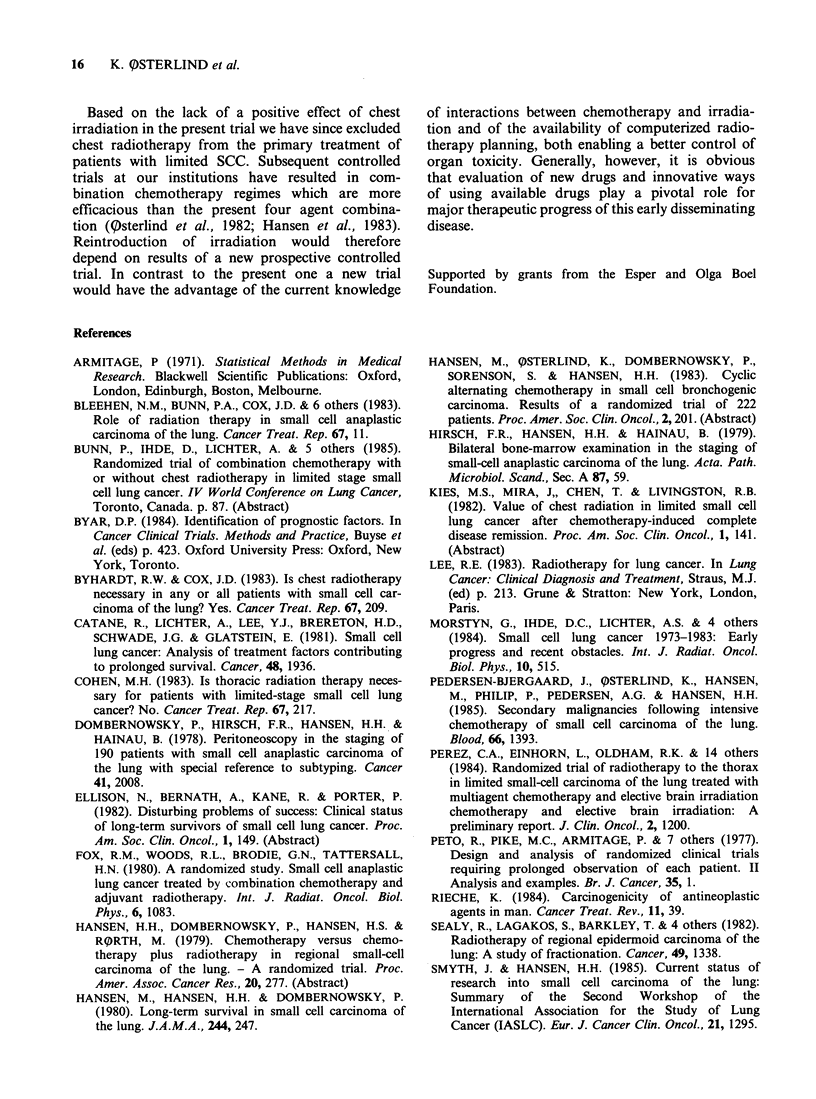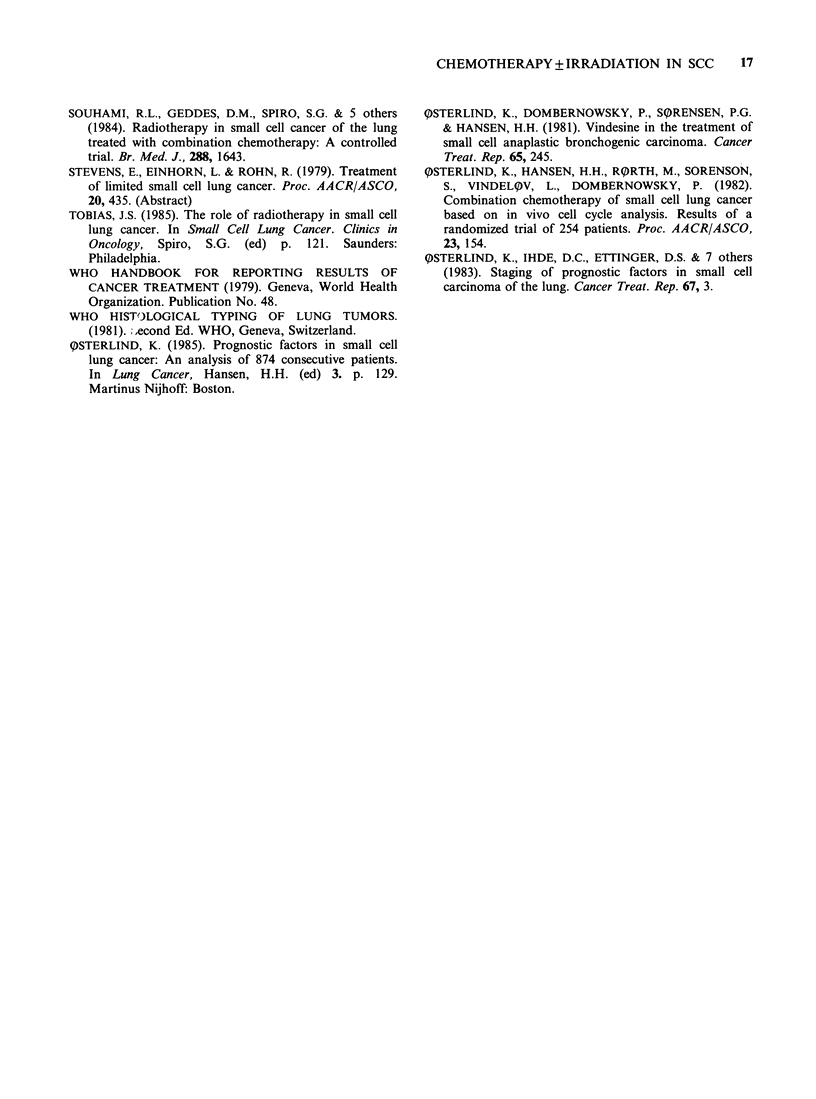# Chemotherapy versus chemotherapy plus irradiation in limited small cell lung cancer. Results of a controlled trial with 5 years follow-up.

**DOI:** 10.1038/bjc.1986.146

**Published:** 1986-07

**Authors:** K. Osterlind, H. H. Hansen, H. S. Hansen, P. Dombernowsky, M. Hansen, M. Rørth

## Abstract

One hundred and forty-five patients with limited stage small cell lung cancer were included in a randomized trial to evaluate the effect of chemotherapy with or without chest irradiation. Seventy-six patients were allotted chemotherapy alone while 69 patients received the same chemotherapy plus radiotherapy, 40 Gy in split-course, administered in weeks 6 and 10 after the initiation of chemotherapy. The chemotherapy consisted of lomustine, cyclophosphamide, vincristine and methotrexate. Patients treated with chemotherapy alone survived for a median of 52 weeks compared to 44 weeks in patients receiving the combined regimen (P = 0.055). After exclusion of five early deaths and one patient refusing the irradiation plus 14 completely resected patients, the remaining 65 patients receiving chemotherapy alone and the 60 patients treated with chemotherapy plus radiotherapy were included in a new analysis. The difference in survival duration which could be ascribed to treatment with or without chest irradiation thereby diminished (P = 0.24). Eighteen months' disease-free survival was obtained in 9.2% of the 65 patients and in 9.8% of the 60 patients. The complete remission rates were 37% and 46%, respectively, (P = 0.33) and the median durations of complete remission were 40 weeks and 52 weeks (P = 0.67). Treatment failure of the primary tumour occurred in 85% of patients treated with chemotherapy alone in contrast to 61% of patients receiving the combined regimen (P = 0.005). Seventy-nine of these patients underwent autopsy at which no residual chest disease was observed in 17% and 37%, respectively (P = 0.045). The combined regimen was more toxic than chemotherapy alone resulting in significantly greater dose reductions and more pronounced thrombocytopenia. Lung and pericardial fibrosis was responsible for four deaths among the complete responders in the radiotherapy group. The combined regimen thus tended to be more efficacious with respect to tumour control at the expense, however, of increased toxicity which per se, eliminated a potential improvement of the overall therapeutical results.


Br. J. Cancer (1986), 54, 7–17

### Chemotherapy *versus* chemotherapy plus irradiation in limited small cell lung cancer. Results of a controlled trial with 5 years follow-up

K. Østerlind[1], H.H. Hansen[1], H.S. Hansen[2], P. Dombernowsky*, M. Hansen[3] & M. Rørth[1]

*Departments of [1]Oncology II; [2]Oncology I, the Finsen Institute, DK-2100 Copenhagen Ø; [3]Medical Department C, Bispebjerg Hospital, DK2400 Copenhagen NV, Denmark.*

**Summary** One hundred and forty-five patients with limited stage small cell lung cancer were included in a randomized trial to evaluate the effect of chemotherapy with or without chest irradiation. Seventy-six patients were allotted chemotherapy alone while 69 patients received the same chemotherapy plus radiotherapy, 40 Gy in split-course, administered in weeks 6 and 10 after the initiation of chemotherapy. The chemotherapy consisted of lomustine, cyclophosphamide, vincristine and methotrexate. Patients treated with chemotherapy alone survived for a median of 52 weeks compared to 44 weeks in patients receiving the combined regimen ($P=0.055$). After exclusion of five early deaths and one patient refusing the irradiation plus 14 completely resected patients, the remaining 65 patients receiving chemotherapy alone and the 60 patients treated with chemotherapy plus radiotherapy were included in a new analysis. The difference in survival duration which could be ascribed to treatment with or without chest irradiation thereby diminished ($P=0.24$). Eighteen months' disease-free survival was obtained in 9.2% of the 65 patients and in 9.8% of the 60 patients. The complete remission rates were 37% and 46%, respectively, ($P=0.33$) and the median durations of complete remission were 40 weeks and 52 weeks ($P=0.67$). Treatment failure of the primary tumour occurred in 85% of patients treated with chemotherapy alone in contrast to 61% of patients receiving the combined regimen ($P=0.005$). Seventy-nine of these patients underwent autopsy at which no residual chest disease was observed in 17% and 37%, respectively ($P=0.045$). The combined regimen was more toxic than chemotherapy alone resulting in significantly greater dose reductions and more pronounced thrombocytopenia. Lung and pericardial fibrosis was responsible for four deaths among the complete responders in the radiotherapy group.

The combined regimen thus tended to be more efficacious with respect to tumour control at the expense, however, of increased toxicity which *per se*, eliminated a potential improvement of the overall therapeutical results.

In contrast to the other main histologic types of lung cancer small cell carcinoma (SCC) is generally non-resectable but highly sensitive to both irradiation and chemotherapy. Irradiation was the main treatment modality of SCC before the introduction of chemotherapy. In the early seventies various combination chemotherapy regimens were developed resulting in prolongation of the survival duration and in a significant, although minute, fraction of disease-free long-term survivors (Hansen *et al.*, 1980; Morstyn *et al.*, 1984). At many centres radiotherapy was maintained as a part of the treatment at least in patients with disease confined to well defined radiation portals. Whether such combined therapy provides advantages compared to chemotherapy alone has been debated for more than a decade and a definite role of chest irradiation in the treatment of SCC has not yet been established (Morstyn *et al.*, 1984; Bleehen *et al.*, 1983; Tobias, 1985; Byhardt & Cox, 1983; Cohen, 1983). The combined modality treatment strategy can be justified by a reduced frequency of chest relapse in irradiated patients, and by results from uncontrolled studies suggesting prolonged survival and more long-term survivors (Byhardt & Cox, 1983). In contrast a strategy based on chemotherapy alone is supported by preliminary results of randomized trials in which the combined regimens did not result in significant advantages in the median survival figures but only additional toxicity, often enhanced by the chemotherapy (Cohen, 1983).

The problem of how best to treat limited stage SCC has not diminished since the present prospective randomized study on chemotherapy with or without chest irradiation was initiated in 1976 (Hansen *et al.*, 1979). The final results of the trial are now available with 5-year follow-up. This is an unusually long period before publication of

Correspondence: H.H. Hansen.
*Current address: Copenhagen University Hospital, Herlev, DK-2730 Herlev, Denmark.
Received 2 January 1986; and in revised form, 7 March 1986.

© The Macmillan Press Ltd., 1986

results from trials on SCC but not disadvantageous as it enables an analysis of overall as well as long-term survival based on complete observations rather than estimates.

### Materials and methods

All patients with SCC referred to the Finsen Institute or Bispebjerg Hospital from May 1976 to January 1979 were included in the trial provided (i) the diagnosis of SCC was histologically or cytologically satisfied the WHO criteria (The World Health Organisation, 1981); (ii) the patient was aged 70 years or less; (iii) the patient had no previous cancer except primary skin tumours (excluding melanomas); (iv) that no chemo- or radiotherapy had been given; and (v) that informed consent was obtained.

Prior to initiation of therapy all patients were evaluated with a routine history and physical examination, chest X-ray, bilateral bone marrow examinations (Hirsch *et al.*, 1979) and peritoneoscopy with liver biopsy (Dombernowsky *et al.*, 1978). Aspirates or biopsies were obtained when pleural fluid, enlarged lymph nodes or cutaneous metastases were observed. Brain scans were only carried out in patients suspected of metastases. Complete blood count, serum concentrations of electrolytes, creatinine, lactic dehydrogenase (LDH), glutamic oxalo acetic transminase, alkaline phosphatase and bilirubin were obtained routinely.

Only patients with limited disease were included, i.e. patients with tumour confined to the primary site and the regional lymph nodes, including the mediastinal and the ipsi- and contralateral supraclavicular nodes (Østerlind *et al.*, 1983). Stratification according to performance status (WHO Handbook for Reporting Results of Cancer Treatment, 1979) was carried out prior to the allotment to chemotherapy with or without chest irradiation.

### *Treatment*

All patients received the same four drug combination chemotherapy consisting of lomustine $70\,mg\,m^{-2}$ p.o., cyclophosphamide $1000\,mg\,m^{-2}$ i.v. and vincristine $1.3\,mg\,m^{-2}$ i.v. (maximally 2 mg) administered on day 1 followed by methotrexate $20\,mg\,m^{-2}$ p.o. on days 15 and 18. This treatment was repeated every 4 weeks. Vincristine $1.3\,mg\,m^{-2}$ was furthermore given on days 8, 15 and 22 in the first cycle. Doses of chemotherapy were increased by 33% if the blood counts remained within normal limits, but were reduced 33% if the WBC count was between 2—$3{,}000\,mm^{-3}$ or the platelet count was 75–$100{,}000\,mm^{-3}$. Treatment was withheld if the WBC count was $<2{,}000\,mm^{-3}$ or the platelet count was $<75{,}000\,mm^{-3}$, and reinstituted when the counts rose above these values.

Irradiation was administered through opposed antero-posterior portals, including the primary tumour, both hili, and the mediastinum, shaped to comprise 1 cm margins of normal lung. The supraclavicular lymph nodes were only included if metastases were suspected. No spinal cord shielding was used. A 6 MV linear accelerator was employed and all patients were treated at the Finsen Institute. A total dose of 40 Gy was delivered in two 5-day series, 4 Gy per daily fraction on days 43–47 and days 71–75. The radiotherapy was thus scheduled to be delivered with the second and third doses of methotrexate. The radiation portals were adjusted before the second series according to radiographic changes of the tumour.

Irradiation as well as chemotherapy was given under out-patient conditions. The chemotherapy was continued until progression or for 18 months, when reevaluation was undertaken, repeating bronchoscopy, bone marrow examination and peritoneoscopy with liver biopsy. Patients having progressive disease received further chemotherapy with alternative agents if their condition permitted while irradiation was only instituted on specific indications such as brain metastases, superior vena caval syndrome or total atelectasis.

### *Evaluation of results*

Duration of survival and survival differences between the two treatment groups were investigated by use of the life-table and the log rank methods (Peto *et al.*, 1977). Test statistics with $P<0.05$ in a two-tailed test were regarded as significant.

Two analyses were carried out. First, survival data on all patients allocated to the two treatment groups were compared. The second analysis focussed more specifically on the intention of the applied treatments and completely resected patients, and patients dying before day 43 were therefore excluded. Evaluation of response, relapse, and toxicity was restricted to the reduced series. It should be noticed that all resected patients and most early deaths would be ineligible for response and occurrence of local relapse, anyway, and that information on toxicity-related early deaths would be irrelevant for the comparison of the two treatment regimens.

The exclusion of patients might result in imbalances between prognostic features of the two treatment groups. Aims to reduce the influence from such imbalances were therefore undertaken according to guidelines described by Peto *et al.* (1977) and Byar (1984). Thus, the patients were stratified into three groups based on pretreatment performance status, serum LDH, and sex, and the survival data were tested again, using the stratified log rank test. The three variables proved to be important prognostic factors in a multivariate analysis including 874 patients (Østerlind, 1985).

Evaluation of response followed the WHO criteria (WHO Handbook for Reporting Results of Cancer Treatment, 1979). All roentgenograms and clinical charts were evaluated retrospectively to obtain as uniform and accurate a determination of response and response duration as possible. Duration of response was counted from the day when at least a partial remission was obtained to reappearance of the disease. In general remissions were not proven before day 28 because chest X-rays were only carried out at 4-week intervals. Exclusion of deaths occurring during the first 42 days eliminated problems related to response classification of early deaths. Death in remission results in incomplete follow-up of the 'real' response duration. The life table and log rank methods were therefore employed for the evaluation of response duration as recommended by the WHO committee (WHO Handbook for Reporting Results of Cancer Treatment, 1979; Peto *et al.,* 1977). Occurrence of brain metastases as the only evidence of recurrent disease, was not regarded as relapse. Deaths in partial remission were classified as progressions on the day of death, as were deaths in complete remission, if residual primary tumour or systemic metastases were proven at a subsequent autopsy. Post mortem examinations were achieved in 68% of the patients.

Differences in response rates between the treatment groups were evaluated for statistical significance by use of the chi square test (Armitage, 1971).

Evaluation of haematologic toxicity was based on nadir values of the haemoglobin concentration, plus WBC and platelet counts as observed on a scheduled day of therapy. Recordings on intervening days were not routine in all patients and such data were therefore omitted from this analysis. The number of doses of cyclophosphamide and the total cumulated dose $m^{-2}$ surface-area were recorded in order to compare dose reductions undertaken in the two treatment groups. Cyclophosphamide was chosen among the four agents in the combination because dose adjustments of this agent, in contrast to lomustine, are not restricted by a fixed content in mg per capsule, and because adjustments of this agent almost exclusively depend on the haematological toxicity. Wilcoxon's rank sum test (Armitage, 1971) was applied to test for differences between the two groups, in nadir values, and in avergae cyclo-phosphamide doses received by each patient.

### Results

A total of 148 patients were included in the trial, 105 at the Finsen Institute and 43 at Bispebjerg Hospital. One patient was lost to follow-up after the initial dose and two patients were excluded at the revision of the histopathological specimens, leaving 145 eligible patients of whom 76 were allotted to chemotherapy alone and 69 to chemo-plus radiotherapy. The diagnosis of SCC was based on cytology alone in five and four of these patients, respectively. Survival curves of the two groups of patients are shown in Figure 1. The difference between the curves was not significant ($P=0.055$), although patients treated with chemotherapy alone generally survived longer than those receiving the combined regimen, the median survival being 52 weeks and 44 weeks, respectively.

**Figure 1** Survival of 145 patients with limited SCC according to whether they were allotted to chemotherapy alone (——; $n=76$) or chemotherapy plus irradiation (----; $n=69$).

Twenty patients were excluded from the subsequent analyses. Eleven and three patients treated with chemotherapy alone or chemo- and radiotherapy, respectively, were excluded because they underwent a complete resection prior to inclusion in the trial. One patient, refusing radiotherapy in spite of initial consent, and 5 early deaths, all occurring in the group allotted to irradiation, were also excluded. Three patients with signs of progressive disease at chest X-ray and one in whom brain metastases appeared during the first

6 weeks of chemotherapy all received the scheduled chest irradiation and remained in the reduced combined treatment group. Remaining after these exclusions were 65 patients treated with chemotherapy alone and the 60 patients receiving the combined regimen. Survival curves are shown in Figure 2. The median survival durations were 50 weeks and 46 weeks, respectively ($P=0.24$).

**Figure 2** Survival of 125 patients eligible for evaluation of treatment efficacy, 65 patients (——) treated with chemotherapy alone and 60 (----) treated with chemotherapy plus irradiation.

A stratified analysis of the survival data was carried out in order to reduce confounding influence from the three main pretreatment prognostic factors (Table I). The lower half of the Table includes the distribution of the patients on prognostic strata and the median survival durations observed within each stratum. Treatment with chemotherapy alone was associated with longest survival duration at all three levels but the effect was not significant ($P=0.53$).

Survival beyond. the 18 months for which chemotherapy was scheduled was observed in 11 patients from each treatment group. Six patients from each group (9.2% *vs*. 9.8%) had no evidence of residual disease at the restaging. Systemic recurrences later appeared in three and brain metastases alone in 2 of the 12 disease-free 18 month survivors. No relationship was apparent between risk of late relapse and type of treatment (Table II).

**Table I** Pretreatment prognostic factors in 125 patients eligible for evaluation of treatment efficacy. The lower part of the table includes a retrospective stratification based on the three factors and median survival durations observed within the strata

| *Prognostic factors and strata* | *Chemotherapy alone n=65* | *Chemo- plus radiotherapy n=60* |
|---|---|---|
| Sex: males | 73% | 72% |
| females | 27% | 28% |
| Performance status: 0 | 35% | 37% |
| 1 | 56% | 53% |
| 2 | 9% | 9% |
| 3–4 | 0% | 1% |
| LDH: normal | 62% | 54% |
| elevated | 38% | 46% |
| Prognosis: | | |
| Good | 43% | 39% |
| Intermediate | 32% | 39% |
| Bad | 25% | 23% |
| Median survival: Prognosis | | |
| Good | 60 weeks | 54 weeks |
| Intermediate | 48 weeks | 44 weeks |
| Bad | 38 weeks | 32 weeks |

*Evaluation of response*

Sixty patients treated with chemotherapy alone and 57 patients receiving the combined regimen were evaluable for response. Three and two patients, respectively, were inevaluable because incomplete resections were carried out prior to the admission, and two and one patients, respectively, had no evaluable lesions on the chest X-ray. Complete remission of all clinical and radiological signs of disease was observed in 22 (37%) of the patients treated with chemotherapy alone, compared to 26 (46%) of those receiving the combined regimen ($P=0.33$). Two of the 26 patients had no signs of response prior to initiation of radiotherapy. Partial remissions were achieved in 27 patients (45%) and 24 patients (42%), respectively. All 24 patients responded to chemotherapy before irradiation was initiated. The overall response rates in the two treatment groups (82% *versus* 88%) were not significantly different ($P=0.38$).

The response duration analysis included 18 complete responders dying without clinical evidence of relapse, 5 treated with chemotherapy alone and 13 receiving the combined regimen. One and 6 of these patients, respectively, had residual or metastatic disease at necropsy (Tables II and III) and were accordingly regarded as recurrences at the day of

**Table II** Outcome of twelve 18-months' disease-free survivors. Survival was counted from initiation of chemotherapy

| *Regimen and no.* | *Sex* | *Outcome* | | *Survival free of SCC (weeks)* | *Autopsy findings* | |
|---|---|---|---|---|---|---|
| | | | | | *Systemic* | *Brain* |
| 1–1 | M | Brain metastases | * | 81 | 0 | + |
| 1–2 | M | Alcoholic liver cirrhosis | * | 131 | 0 | – |
| 1–3 | M | Recurrent SCC | | 186 | – | – |
| 1–4 | F | Cardiac disease | * | 201 | – | – |
| 1–5 | F | Adenocarcinoma in the contralateral lung | * | 260 | 0 | 0 |
| 1–6 | M | Alive and disease-free | | 451 | – | – |
| 2–1 | M | Radiation lung fibrosis | * | 84 | 0 | 0 |
| 2–2 | F | Radiation lung + pericardial fibrosis | * | 89 | 0 | 0 |
| 2–3 | M | Recurrent SCC | | 99 | + | 0 |
| 2–4 | M | Recurrent SCC | | 118 | + | + |
| 2–5 | F | Brain relapse | * | 259 | 0 | + |
| 2–6 | M | Malignant astrocytoma | * | 281 | 0 | 0 |

Regimen: 1: chemotherapy alone, 2: chemo- plus radiotherapy. Sex: M: male, F: female. Autopsy findings: 0: no SCC, +: SCC, –: not examined. *: Died in clinical systemic complete remission.

**Table III** Causes of death and autopsy findings in 10 complete responders dying without clinical signs of systemic recurrence while still receiving chemotherpy

| *Regimen and no.* | *Sex* | *Cause of death* | *Survival (weeks)* | *Autopsy findings* | |
|---|---|---|---|---|---|
| | | | | *Systemic* | *Brain* |
| 1–1 | M | Pneumonia | 18 | + | 0 |
| 2–1 | F | Fungal pneumonia | 21 | + | 0 |
| 2–2 | M | Pneumonia | 24 | 0 | – |
| 2–3 | M | Brain metastases | 28 | + | + |
| 2–4 | M | Myocardial infarction | 30 | 0 | 0 |
| 2–5 | M | Radiation pericarditis | 48 | 0 | 0 |
| 2–6 | M | Brain metastases | 49 | – | – |
| 2–7 | F | Radiation lung fibrosis | 50 | + | 0 |
| 2–8 | M | Brain metastases | 56 | + | + |
| 2–9 | M | Brain metastases | 58 | – | – |

Regimen: 1: chemotherapy alone, 2: chemo- plus radiotherapy. Sex: M: male, F: female. Autopsy findings: 0: no SCC, +: SCC, –: not examined.

death. Two patients with clinically verified brain metastases (Nos. 2–6 and 2–9, Table III) had no necropsy and were categorized as relapses because systemic disease was proven post mortem in two comparable cases (Nos. 2–3 and 2–8). The resulting life tables of complete and partial remission duration in the two treatment groups are shown in Figure 3. The response durations tended to be longest in patients receiving the combined regimen but the differences were not significant for complete ($P = 0.67$) or partial ($P = 0.58$) responders. The median values were 40 weeks and 46 weeks for the duration of complete remission and 22 weeks and 26 weeks for partial remissions, corresponding to patients treated with chemotherapy alone or with the combined treatment regimen, respectively.

*Relapse pattern*

Clinical evidence of disease progression was

**Figure 3** Life table estimates of remission durations in complete responders (upper curves), 22 treated with chemotherapy alone (——) and 26 receiving chemotherapy plus irradiation (----). The lower curves show the response duration in partial responders, 27 patients treated with chemotherapy alone (——) and 24 (----) patients receiving chemotherapy plus irradiation.

achieved in 52 (87%) and 37 (65%) of the 60 and 57 patients who were evaluable for response. Three (5%) and seven (12%) patients, respectively, had stable partial tumour remissions at the time of death. Radiologic evidence of intrathoracic progression, alone or in combination with occurrence

**Table IV** Clinical disease status at time of death in patients with evaluable tumours

| | *Chemotherapy alone n=60* | *Chemotherapy+ irradiation n=57* |
|---|---|---|
| *No progression of systemic disease:* | | |
| Complete responders[a] | 5 | 13 |
| Partial responders | 3 | 7 |
| *Progression:* | | |
| Intrathoracic recurrence | 48 | 28 |
| Extrathoracic recurrence only | 4 | 9 |

[a]Additional data on these patients are given in **Tables II and III.**

of distant metastases, was observed in 48 and 28 patients, respectively. Evidence of remaining or recurrent tumour at chest X-ray was thus present in 51 (85%) and 35 (61%) of the patients at the time of death ($P=0.005$) (Table IV).

Thirty-six (60%) and 43 (75%) of the 117 evaluable patients underwent post mortem examinations (Table V). No residual primary tumour was observed in 10 (28%) of the patients receiving chemotherapy alone compared to 24 (56%) of the irradiated patients ($P=0.013$). Four and 8 of these patients, respectively, had residual SCC in the chest, outside the primary lung. Control of chest disease was thus achieved in 6 (17%) and 16 (37%) of the patients, respectively ($P=0.045$).

**Table V** Postmortem findings in 79 (68%) of the 117 evaluable patients

| | *A: Chemotherapy alone n=36* | *B: Chemotherapy +irradiation n=43* | *A vs B P* |
|---|---|---|---|
| No residual SCC | 2 (8%) | 8 (23%) | 0.08 |
| Only brain metastases | 1 | 2 | |
| Residual systemic disease | 33 | 33 | |
| Sites: | | | |
| Residual primary tumour | 26 (79%) | 19 (58%) | 0.07 |
| Residual tumour in chest | 30 (91%) | 27 (82%) | 0.3 |
| Liver metastases | 23 (70%) | 22 (67%) | 0.8 |
| Adrenal metastases | 9 (27%) | 12 (36%) | 0.4 |
| Brain metastases[a] | 11/26 (42%) | 14/32 (44%) | 0.9 |

[a]Including the three patients with autopsy findings of SCC confined to the brain.

*Toxicity of therapy*

Fifty of the 60 patients treated with the combined regimen received both series of irradiation. One patient refused the second course while 9 patients died or progressed during the three weeks rest period.

Equal numbers of chemotherapy courses were administered in the two treatment groups but dose reductions were greatest in the irradiated patients. A median of 7 doses of cyclophosphamide was thus given to each patient in both groups, the ranges being 3–17 and 2–19, respectively. The average dosage of cyclophosphamide was 790 mg $m^{-2}$ $dose^{-1}$ (range: 500–1050) in patients treated with chemotherapy alone, and 710 mg $m^{-2}$ $dose^{-1}$ (range: 360–980) in the irradiated patients ($P = 0.05$).

Dysphagia for one to two weeks after radiotherapy was recorded in 44 patients (72%) while this symptom was not registered in non-irradiated patients. Roentgenographic signs of pulmonary fibrosis within the radiation portals were observed in 26 patients (43%). Respiratory insufficiency and pericardial effusion (in two patients) were the main causes of death in 4 complete responders (Tables II and III), and an emergency pericardiotomy was carried out in one patient (No. 2–6, Table II) 71 weeks after initiation of the radiotherapy.

The haematologic toxicity observed in the two treatment groups is summarized in Table VI. The degree of thrombocytopenia was significantly greater in the irradiated patients compared to those receiving chemotherapy alone, while haemoglobin and leukocyte nadir values did not differ significantly between the groups. Nine and 5 patients, respectively, were hospitalized and treated with i.v. antibiotics because of febrile episodes during leukopenia. Two patients, both receiving the combined regimen, died at such incidents.

Hospital admissions were undertaken with the same frequency in the two treatment groups, the median duration of hospitalization corresponded to 3% of the treatment duration in both groups.

*Chemotherapy after relapse.*

Fifty of the 52 patients relapsing after treatment with chemotherapy alone and 30 of the 37 recurrences observed in the irradiated group received a two-agent combination of etoposide 100 mg $m^{-2}$ p.o. for 4 days and doxorubicin 30 mg $m^{-2}$ i.v., repeated every 3 weeks. Forty-four and 18 of these patients were evaluable for response. Objective remissions were observed in 6 and 2 patients, respectively, corresponding to a response rate of 13%. Response duration was short with a median of 6 weeks and a range of 5–42 weeks.

Subsequent chemotherapy with investigative agents in phase II trials were given to 21 and 13 patients from the respective treatment groups. Ten and five of these patients received vindesine and partial remissions were observed in 3 and one patients, respectively, the response duration being 4 to 7 weeks (Østerlind *et al.*, 1981).

**Table VI** Haematological toxicity. Proportions of patients experiencing nadir values (on days when therapy was scheduled) within the signified intervals, at least once in the treatment

| | *A: Chemotherapy alone n=65* | *B: Chemotherapy + irradiation n=61* | *A vs. B*[a] *P* |
|---|---|---|---|
| Haemoglobin (g $dl^{-1}$) | | | |
| > 11.0 | 25% | 18% | |
| 9.6–11.0 | 48% | 39% | 0.15 |
| 8.1–9.5 | 20% | 36% | |
| ≦ 8.0 | 7% | 7% | |
| Leukocytes ($10^3$ $mm^{-3}$) | | | |
| ≧ 3.0 | 6% | 5% | |
| 2.0–2.9 | 17% | 11% | 0.38 |
| 1.0–1.9 | 49% | 54% | |
| < 1.0 | 28% | 30% | |
| Platelets ($10^3$ $mm^{-3}$) | | | |
| ≧ 100 | 55% | 36% | |
| 50–99 | 28% | 28% | 0.01 |
| 0–49 | 17% | 36% | |

[a]Result of Wilcoxon's rank sum test.

**Discussion**

The question whether or not thoracic irradiation adds to the results of chemotherapy in patients with limited stage SCC has been unanswered for more than a decade. Data from non-randomized trials have suggested that there may be some advantages of combined modality therapy but valid conclusions generally cannot be drawn from uncontrolled studies. Within the last five years preliminary data from randomized trials have been published. Available information from ongoing trials, were discussed and evaluated at the First International Workshop on Small Cell Lung Cancer in 1981, but definitive answers could not be established (Bleehen *et al.*, 1983). Updated and more detailed data from a number of these studies are now available (Table VII) (Stevens *et al.*, 1979; Fox *et al.*, 1980; Kies *et al.*, 1982; Bunn *et al.*, 1985; Souhami *et al.*, 1984; Perez *et al.*, 1984; Smyth & Hansen, 1985). Whether chemotherapy with or without thoracic irradiation was employed did not result in significantly different survival durations in the majority of the trials. The combined modality therapy appeared to be significantly better than chemotherapy alone in two of the studies but further follow-up of these preliminary data should be awaited before too firm conclusions are drawn. The survival data and especially the long-term results reported in the present study are definitive while projected two-year survival rates, such as those estimated in three of the studies, may have a tendency to change with further follow-up (Cohen, 1983). This tendency may predominate in irradiated patients due to late radiation toxicity as observed in this as well as in other studies of long survivors (Catane *et al.*, 1981; Ellison *et al.*, 1982). At present the total available number of two-year survivors is still too small to enable definite conclusions about a possible advantageous role of chest irradiation in long-term control of limited SCC.

The combined modality regimen employed in the present trial resulted in marginally more complete remissions and longer complete response durations than when chemotherapy was used alone. In addition a higher fraction of patients receiving combined modality treatment was disease-free at autopsy. These results were obtained at the expense of extra haematologic toxicity in spite of an average 10% dose reduction, and the question arises whether or not the same degree of disease control could have been achieved with an equitoxic chemotherapy regimen. More intensive chemotherapy would presumably result in more treatment related deaths including a slightly increased risk of secondary leukaemia (Pedersen-Bjergaard *et al.*, 1985; Rieche, 1984), but the long-term results would not be influenced by morbidity and mortality due to irradiation pulmonary and pericardial fibrosis (Ellison *et al.*, 1982).

The radiotherapy regimen employed in the present study was effective in controlling the primary tumour as reflected by a significantly reduced frequency of residual chest disease at autopsy, but the efficacy was obtained at the cost of a number of deaths in complete remission. The present irradiation regimen was widely used at the time the trial was planned. Thus, it was the current treatment of potentially curable lung cancer at the Mayo Clinic (Lee, 1983). The side effects of this short course radiation regimen were compared to those of a less intensive schedule, delivering 50 Gy in 15 fractions in 3 weeks plus 10 fractions in 2 weeks, with 2 weeks rest (Sealy *et al.*, 1982). The toxicity was acceptable in both treatment groups and no significant differences were observed. Knowledge about potentiation between irradiation and chemotherapy has increased considerably during the last decade and several of the irradiation regimens listed in Table VII may therefore be more appropriate for combined therapy. More long-term results, especially from randomized trials, should be accrued, however, before evaluation of advantages *versus* disadvantages of the different regimens is carried out.

It has been discussed whether radiotherapy should be administered early or late in the treatment course. A treatment policy of late irradiation restricted to responders does not find support in available data (Fox *et al.*, 1980; Bunn *et al.*, 1985) (Table VII) and treatment of all patients concurrently with the first dose of chemotherapy or 4, 9 or 12 weeks later does not seem to result in major differences. Late addition of the radiotherapy is, however, associated with an often overlooked methodologic enigma. If randomization is carried out on the first day of chemotherapy, as it was in all trials summarized in Table VII, the risk of accidentally occurring differences between the treatment groups will increase with time until initiation of radiotherapy. About one fifth of the patients in the British study (Souhami *et al.*, 1984) thus did not meet the criteria to receive radiotherapy at time of reevaluation, 12 weeks after allotment, and in the trial from the Southeastern Cancer Study Group (Peréz *et al.*, 1984) 11% of the patients randomized to receive chemotherapy alone and 5% of the combined modality treated group either expired or had disease progression before day 29 when chest irradiation was initiated. This problem should be avoided in future trials although retrospective adjustments, as carried out in the present analysis, may enable a more accurate evaluation of the effect, ascribed to different efficacies of the investigated treatment regimens.

**Table VII** Randomized trials of chemotherapy *vs.* chemotherapy plus radiotherapy in limited stage SCC

| Investigator | | *Chemo- and radiotherapy* | | | | | *No. of pts.* | | *Median survival (weeks)* | | | | *2 year disease-free survivors* | |
|---|---|---|---|---|---|---|---|---|---|---|---|---|---|---|
| | | | | | | | | | | | | | *Actual or projected %* | |
| | | *Drugs* | *Dose* | *Fractions* | *First week* | *PCI* | *CT* | *CT+RT* | *CT* | *CT+RT* | *log-rank test* | *Toxicity** | *CT* | *CT+RT* |
| Stevens, | 1979 | CAV | 35 Gy split | 15 | 4 | + | 18 | 14 | 50 | 56 | $P>0.05$ | — | — | — |
| Fox, | 1980[1] | CAV | 40 Gy | 20 | 9 | – | 44 | 40 | 55 | 72 | $P=0.003$[2] | — | 1/44 | 6/40 |
| Kies, | 1982 | MEV~ CAV | 48 Gy split | 22 | 12 | + | 21[3] | 28[3] | 49 | 55 | $P=0.2$ | — | — | — |
| Bunn, | 1985 | CML~ VAP | 40 Gy | 15 | 1 | + | 44 | 44 | 52 | 65 | $P<0.06$ | */** | 10% | 30% |
| Souhami, | 1984 | AV~ CM | 40 Gy | 20 | 12 | – | 73 | 57 | R: 52<br>N: 29 | 57<br>35[4] | $P>0.05$<br>$P>0.05$ | */*<br>*/* | 12%<br>12% | 14%<br>4% |
| Perez, | 1984 | CAV >wk18:HE | 36 Gy split | 15 | 5 | + | 142 | 149 | 49 | 60 | $P=0.03$ | */* | 19% | 28% |
| Present trial | | CMVL | 40 Gy split | 10 | 6 | – | 65[5] | 60[5] | 50 | 46 | $P=0.24$ | */** | 5/65 | 2/60 |

A: Doxorubicin. C: Cyclophosphamide. E: Etoposide. H: Hexamethylmelamine. L: Lomustine. M: Methotrexate. P: Procarbazine. V: Vincristine. PCI: Prophylactic cranial irradiation. CT: Chemotherapy. RT: Radiotherapy. ~: Alternating with. *: * = grade 0–2, ** = grade 2–4. R: Responders. N: Non responders. 1: Updated results from the Second Workshop on Small Cell Lung Cancer, 1984 (Smyth & Hansen, 1985). 2: Significance of the estimated influence of radiotherapy in a multivariate regression analysis. 3: Only complete responders. 4: Only patients with stable disease received irradiation. 5: Deaths before radiotherapy and completely resected patients were excluded.

Based on the lack of a positive effect of chest irradiation in the present trial we have since excluded chest radiotherapy from the primary treatment of patients with limited SCC. Subsequent controlled trials at our institutions have resulted in combination chemotherapy regimes which are more efficacious than the present four agent combination (Østerlind *et al.*, 1982; Hansen *et al.*, 1983). Reintroduction of irradiation would therefore depend on results of a new prospective controlled trial. In contrast to the present one a new trial would have the advantage of the current knowledge of interactions between chemotherapy and irradiation and of the availability of computerized radiotherapy planning, both enabling a better control of organ toxicity. Generally, however, it is obvious that evaluation of new drugs and innovative ways of using available drugs play a pivotal role for major therapeutic progress of this early disseminating disease.

Supported by grants from the Esper and Olga Boel Foundation.

**References**

ARMITAGE, P (1971). *Statistical Methods in Medical Research*. Blackwell Scientific Publications: Oxford, London, Edinburgh, Boston, Melbourne.

BLEEHEN, N.M., BUNN, P.A., COX, J.D. & 6 others (1983). Role of radiation therapy in small cell anaplastic carcinoma of the lung. *Cancer Treat. Rep.* **67,** 11.

BUNN, P., IHDE, D., LICHTER, A. & 5 others (1985). Randomized trial of combination chemotherapy with or without chest radiotherapy in limited stage small cell lung cancer. *IV World Conference on Lung Cancer,* Toronto, Canada. p. 87. (Abstract)

BYAR, D.P. (1984). Identification of prognostic factors. In *Cancer Clinical Trials. Methods and Practice,* Buyse *et al.* (eds) p. 423. Oxford University Press: Oxford, New York, Toronto.

BYHARDT, R.W. & COX, J.D. (1983). Is chest radiotherapy necessary in any or all patients with small cell carcinoma of the lung? Yes. *Cancer Treat. Rep.* **67,** 209.

CATANE, R., LICHTER, A., LEE, Y.J., BRERETON, H.D., SCHWADE, J.G. & GLATSTEIN, E. (1981). Small cell lung cancer: Analysis of treatment factors contributing to prolonged survival. *Cancer,* **48,** 1936.

COHEN, M.H. (1983). Is thoracic radiation therapy necessary for patients with limited-stage small cell lung cancer? No. *Cancer Treat. Rep.* **67,** 217.

DOMBERNOWSKY, P., HIRSCH, F.R., HANSEN, H.H. & HAINAU, B. (1978). Peritoneoscopy in the staging of 190 patients with small cell anaplastic carcinoma of the lung with special reference to subtyping. *Cancer* **41,** 2008.

ELLISON, N., BERNATH, A., KANE, R. & PORTER, P. (1982). Disturbing problems of success: Clinical status of long-term survivors of small cell lung cancer. *Proc. Am. Soc. Clin. Oncol.,* **1,** 149. (Abstract)

FOX, R.M., WOODS, R.L., BRODIE, G.N., TATTERSALL, H.N. (1980). A randomized study. Small cell anaplastic lung cancer treated by combination chemotherapy and adjuvant radiotherapy. *Int. J. Radiat. Oncol. Biol. Phys.,* **6,** 1083.

HANSEN, H.H., DOMBERNOWSKY, P., HANSEN, H.S. & RØRTH, M. (1979). Chemotherapy versus chemotherapy plus radiotherapy in regional small-cell carcinoma of the lung. – A randomized trial. *Proc. Amer. Assoc. Cancer Res.,* **20,** 277. (Abstract)

HANSEN, M., HANSEN, H.H. & DOMBERNOWSKY, P. (1980). Long-term survival in small cell carcinoma of the lung. *J.A.M.A.,* **244,** 247.

HANSEN, M., ØSTERLIND, K., DOMBERNOWSKY, P., SORENSON, S. & HANSEN, H.H. (1983). Cyclic alternating chemotherapy in small cell bronchogenic carcinoma. Results of a randomized trial of 222 patients. *Proc. Amer. Soc. Clin. Oncol.,* **2,** 201. (Abstract)

HIRSCH, F.R., HANSEN, H.H. & HAINAU, B. (1979). Bilateral bone-marrow examination in the staging of small-cell anaplastic carcinoma of the lung. *Acta. Path. Microbiol. Scand.,* Sec. A **87,** 59.

KIES, M.S., MIRA, J,, CHEN, T. & LIVINGSTON, R.B. (1982). Value of chest radiation in limited small cell lung cancer after chemotherapy-induced complete disease remission. *Proc. Am. Soc. Clin. Oncol.,* **1,** 141. (Abstract)

LEE, R.E. (1983). Radiotherapy for lung cancer. In *Lung Cancer: Clinical Diagnosis and Treatment,* Straus, M.J. (ed) p. 213. Grune & Stratton: New York, London, Paris.

MORSTYN, G., IHDE, D.C., LICHTER, A.S. & 4 others (1984). Small cell lung cancer 1973–1983: Early progress and recent obstacles. *Int. J. Radiat. Oncol. Biol. Phys.,* **10,** 515.

PEDERSEN-BJERGAARD, J., ØSTERLIND, K., HANSEN, M., PHILIP, P., PEDERSEN, A.G. & HANSEN, H.H. (1985). Secondary malignancies following intensive chemotherapy of small cell carcinoma of the lung. *Blood,* **66,** 1393.

PEREZ, C.A., EINHORN, L., OLDHAM, R.K. & 14 others (1984). Randomized trial of radiotherapy to the thorax in limited small-cell carcinoma of the lung treated with multiagent chemotherapy and elective brain irradiation chemotherapy and elective brain irradiation: A preliminary report. *J. Clin. Oncol.,* **2,** 1200.

PETO, R., PIKE, M.C., ARMITAGE, P. & 7 others (1977). Design and analysis of randomized clinical trials requiring prolonged observation of each patient. II Analysis and examples. *Br. J. Cancer,* **35,** 1.

RIECHE, K. (1984). Carcinogenicity of antineoplastic agents in man. *Cancer Treat. Rev.,* **11,** 39.

SEALY, R., LAGAKOS, S., BARKLEY, T. & 4 others (1982). Radiotherapy of regional epidermoid carcinoma of the lung: A study of fractionation. *Cancer,* **49,** 1338.

SMYTH, J. & HANSEN, H.H. (1985). Current status of research into small cell carcinoma of the lung: Summary of the Second Workshop of the International Association for the Study of Lung Cancer (IASLC). *Eur. J. Cancer Clin. Oncol.,* **21,** 1295.

SOUHAMI, R.L., GEDDES, D.M., SPIRO, S.G. & 5 others (1984). Radiotherapy in small cell cancer of the lung treated with combination chemotherapy: A controlled trial. *Br. Med. J.*, **288**, 1643.

STEVENS, E., EINHORN, L. & ROHN, R. (1979). Treatment of limited small cell lung cancer. *Proc. AACR/ASCO*, **20**, 435. (Abstract)

TOBIAS, J.S. (1985). The role of radiotherapy in small cell lung cancer. In *Small Cell Lung Cancer. Clinics in Oncology*, Spiro, S.G. (ed) p. 121. Saunders: Philadelphia.

WHO HANDBOOK FOR REPORTING RESULTS OF CANCER TREATMENT (1979). Geneva, World Health Organization. Publication No. 48.

WHO HISTOLOGICAL TYPING OF LUNG TUMORS. (1981). Second Ed. WHO, Geneva, Switzerland.

ØSTERLIND, K. (1985). Prognostic factors in small cell lung cancer: An analysis of 874 consecutive patients. In *Lung Cancer*, Hansen, H.H. (ed) **3**. p. 129. Martinus Nijhoff: Boston.

ØSTERLIND, K., DOMBERNOWSKY, P., SØRENSEN, P.G. & HANSEN, H.H. (1981). Vindesine in the treatment of small cell anaplastic bronchogenic carcinoma. *Cancer Treat. Rep.* **65**, 245.

ØSTERLIND, K., HANSEN, H.H., RØRTH, M., SORENSON, S., VINDELØV, L., DOMBERNOWSKY, P. (1982). Combination chemotherapy of small cell lung cancer based on in vivo cell cycle analysis. Results of a randomized trial of 254 patients. *Proc. AACR/ASCO*, **23**, 154.

ØSTERLIND, K., IHDE, D.C., ETTINGER, D.S. & 7 others (1983). Staging of prognostic factors in small cell carcinoma of the lung. *Cancer Treat. Rep.* **67**, 3.